# Clinical Outcome of Neer Type II Lateral End Clavicle Fractures With Coracoclavicular Ligament Disruption Treated With Pre-Contoured Locking Plate and Endobutton

**DOI:** 10.7759/cureus.12585

**Published:** 2021-01-08

**Authors:** Vikas Vikas, Naval Bhatia, Divesh Jalan, Jatin Prakash, Jujhar Singh, Shailendra Khare

**Affiliations:** 1 Central Institute of Orthopedics, Vardhman Mahavir Medical College and Safdarjang Hospital, New Delhi, IND

**Keywords:** lateral end clavicle fractures, neer type ii, pre-contoured locking plate, coracoclavicular reconstruction, endobutton

## Abstract

Introduction

Many surgical techniques have been described for the treatment of Neer type II lateral end clavicle fractures like open reduction and internal fixation with hook plate, tension band wiring, coracoclavicular screw fixation, and distal clavicle locking plate. However, most of these operative procedures are associated with high perioperative complications ranging from hardware prominence, hardware failure, screw and plate pull-out, and infection. As the lateral end clavicle fractures has both vertical and horizontal stress forces, any technique counteracting both the forces should result in a better clinical outcome. Therefore, this study was conducted to assess the functional and radiological outcome of type II lateral end clavicle fracture treated using pre-contoured locking plate along with coracoclavicular reconstruction with endobutton and fiberwire.

Methods

Thirty-two consecutive patients with Neer type II fractures of the lateral end of clavicle were treated surgically using pre-contoured locking plate and coracoclavicular reconstruction with endobutton and fiberwire between May 2014 and December 2016. Clinical outcome was assessed using the University of California Los Angeles (UCLA) shoulder score and Constant Murley score. The coracoclavicular distance was also recorded. These were compared to the unaffected side at one-year follow-up.

Results

The bony union was achieved in all cases. There were no major complications in any of the patients. All the patients were able to return to their preinjury level of activity. The UCLA score, the Constant Murley score, and coracoclavicular distance did not vary significantly at a one-year interval when compared to the normal shoulder.

Conclusion

Open reduction and internal fixation of Neer type II lateral end clavicle fractures using pre-contoured locking distal clavicle plate along with coracoclavicular reconstruction with endobutton and No. 2 fiberwire provide an excellent functional and radiological outcome.

## Introduction

The clavicle fractures are relatively common injuries because of their subcutaneous position and they constitute 2-4% of all adult fractures [1]. Of these, lateral end clavicle fractures account for 12-15% [2]. Neer divided fractures of the lateral clavicle into three types depending upon the relationship of the fracture line to the coracoclavicular ligaments and acromioclavicular joint [3]. Type I fractures are lateral to the coracoclavicular attachment and are considered stable fractures. Type II fractures are unstable fractures due to disruption of the coracoclavicular ligaments while type III fractures are those fractures where the fracture extends to the acromioclavicular joint and they are again considered stable.

Type I and III fractures are usually minimally displaced and when managed conservatively, the functional results are generally satisfactory [1-3]. On the other hand, type II fractures are unstable because of loss of coracoclavicular ligamentous restraint on the medial fragment [3,4]. In addition, the weight of the upper limb and pull of the latissimus dorsi, pectoralis muscles, and scapular rotators pulls the lateral fragment downwards while the trapezius muscle pulls the medial fragment upwards causing the fracture to displace [5,6]. Non-union rates are as high as 30% in type II fractures because of these deforming forces [3]. The non-union can result in persistent pain, restriction of shoulder movements, and loss of shoulder strength [3,7]. As the management of an established non-union may be technically challenging, open reduction and internal fixation are often recommended as the primary treatment for these unstable fractures [8].

Many operative techniques have been described for type II lateral end clavicle fractures including open reduction and internal fixation with hook plates, tension band wiring, coracoclavicular screw fixation, and distal clavicle locking plates [2,9]. However, these operative procedures are associated with high perioperative complications ranging from hardware prominence, hardware failure, screw and plate pull-out, and infection. When pre-contoured distal locking plates are used without coracoclavicular reconstruction in Neer type II fractures, the stability is less as compared to cases where they are used along with coracoclavicular reconstruction [10]. This is probably because pre-contoured distal locking plates with coracoclavicular reconstruction tends to neutralize both horizontal and vertical deforming forces acting at the fracture site more effectively, thereby reducing complications and providing better functional outcomes. We, therefore, hypothesized that lateral end clavicle fractures when treated with open reduction and internal fixation with pre-contoured locking distal clavicle plate along with coracoclavicular reconstruction with endobutton and No. 2 fiberwire should provide good functional results with reduced complication rates.

The purpose of this study is to assess the functional and radiological outcome of type II lateral end clavicle fracture treated with pre-contoured distal locking plate along with coracoclavicular reconstruction.

## Materials and methods

**Figure 1:**
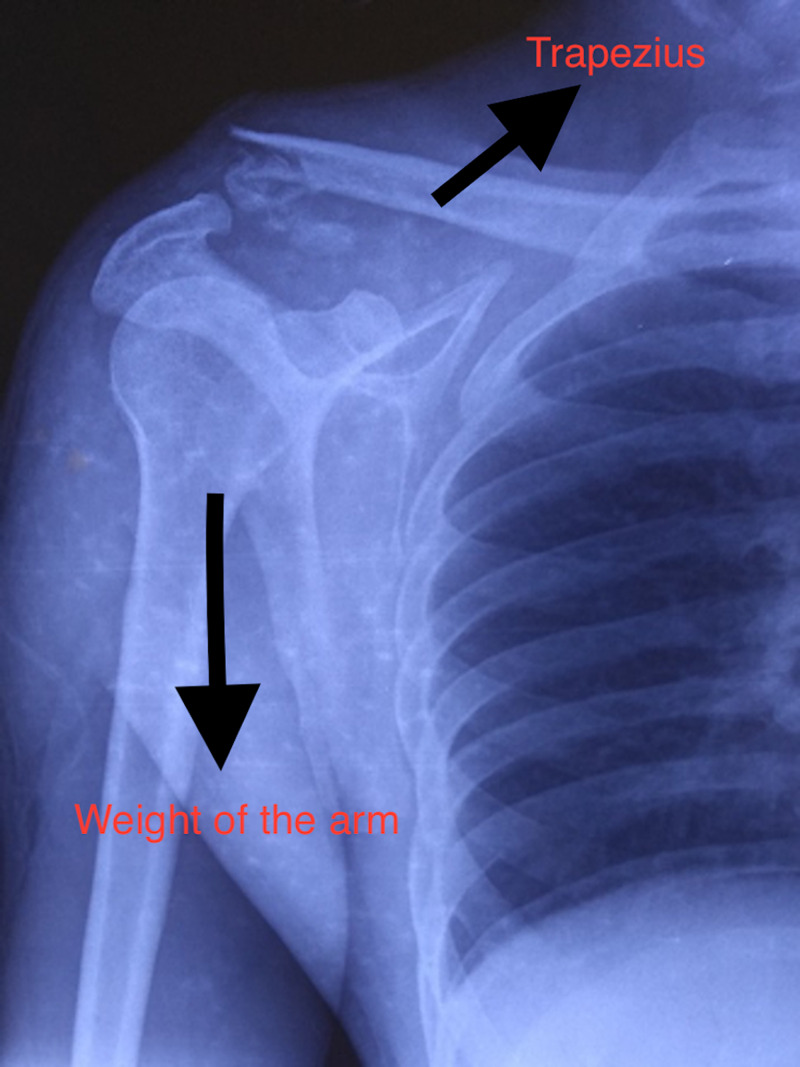
Pre-operative skiagram showing Neer type II lateral end clavicle fracture with displacing forces.

This was a prospective study done at a tertiary care center between May 2014 and December 2016 after obtaining ethical clearance from the Institutional Review Board. All patients with acute unilateral Neer type II fractures in the age group 18-60 years with normal pre-injury shoulder function were included in the study. The patients with associated acromioclavicular joint dislocation, previous history of fracture to the affected shoulder, polytrauma, associated neurovascular injury, pathological fracture, and open fracture were excluded from the study. After informed consent, 32 patients were included in the study. All patients underwent clinical examination and radiological evaluation with plain radiography before surgery (Figure 1). The stress x-ray may help classify these fractures but as the procedure was painful, it was not done.

Surgical procedure

**Figure 2:**
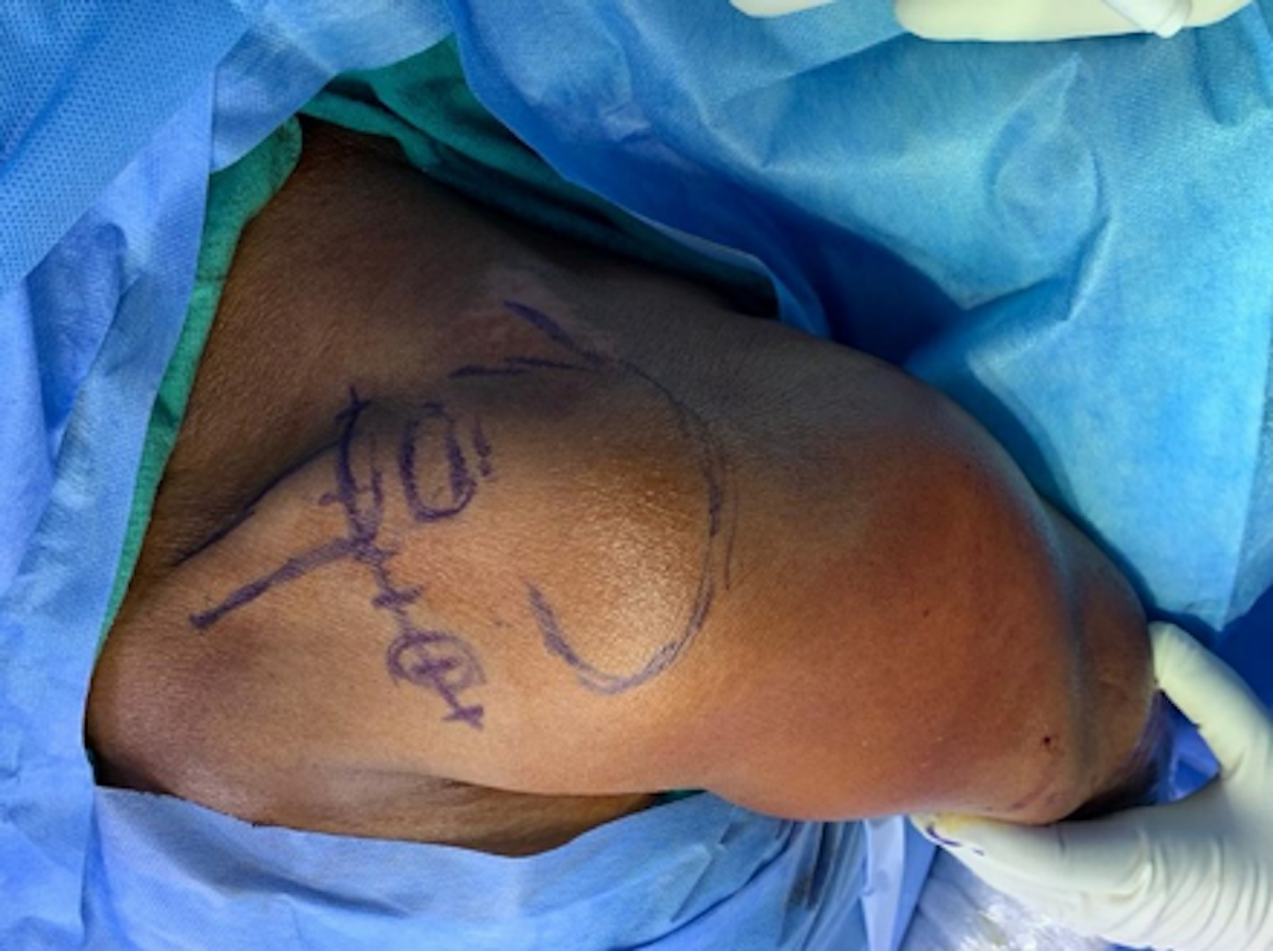
‘Bra strap’ incision from the tip of the coracoid process to anterosuperior aspect of the lateral third of the clavicle.

All the patients were operated on under general anesthesia in a beach chair position. To minimize bleeding, the line of skin incision was infiltrated with 1:200000 epinephrine. A 5-7cm vertical (‘bra-strap’) incision was made from the tip of the coracoid process to the anterosuperior aspect of the lateral third of the clavicle (Figure 2).

Medial and lateral flaps were developed. The deltotrapezial fascia was then incised perpendicular to the skin incision and released from the clavicle. The fracture ends were then exposed. The coracoid was also exposed. Dissection medial to the coracoid was avoided to prevent inadvertent damage to neurovascular structures. A tunnel was made at the coracoid base centrally about 1.5 cm from its tip using a 2.5mm drill bit. Another bony tunnel was made in the clavicle just above the coracoid slightly anterior to the midline. Then, an endobutton (12 mm x 4 mm, Arthrex) loaded with a single No. 2 fiberwire 38" suture was passed under the coracoid and the clavicle tunnel in a retrograde fashion so that the endobutton catches on the undersurface of the coracoid and two ends of the suture come out of the clavicle which can be later tied to the distal clavicle plate. The fracture ends were reduced and fixed with a pre-contoured locking distal clavicle plate. When at least one screw was applied on either side of the fracture the two ends of the suture were tied with the distal clavicle plate. At least three screws (3.5mm cortical/locking) were applied medial to the fracture and three to four screws (2.7 mm locking) lateral to the fracture. Proper hemostasis was achieved and the wound was closed in layers. No drain was applied. The arm was immobilized in an arm pouch.

Post-operatively intravenous antibiotics were given for two days and the patients were discharged on the third day. Gentle pendular exercises were started on the third day and passive shoulder range of motion (ROM) exercises on the seventh day with restriction of shoulder abduction to 90 degrees. The assisted active motion was started at two weeks after suture removal. The patients were allowed both active and passive full range of movements after four weeks. The arm pouch was removed after six weeks. 

**Figure 3:**
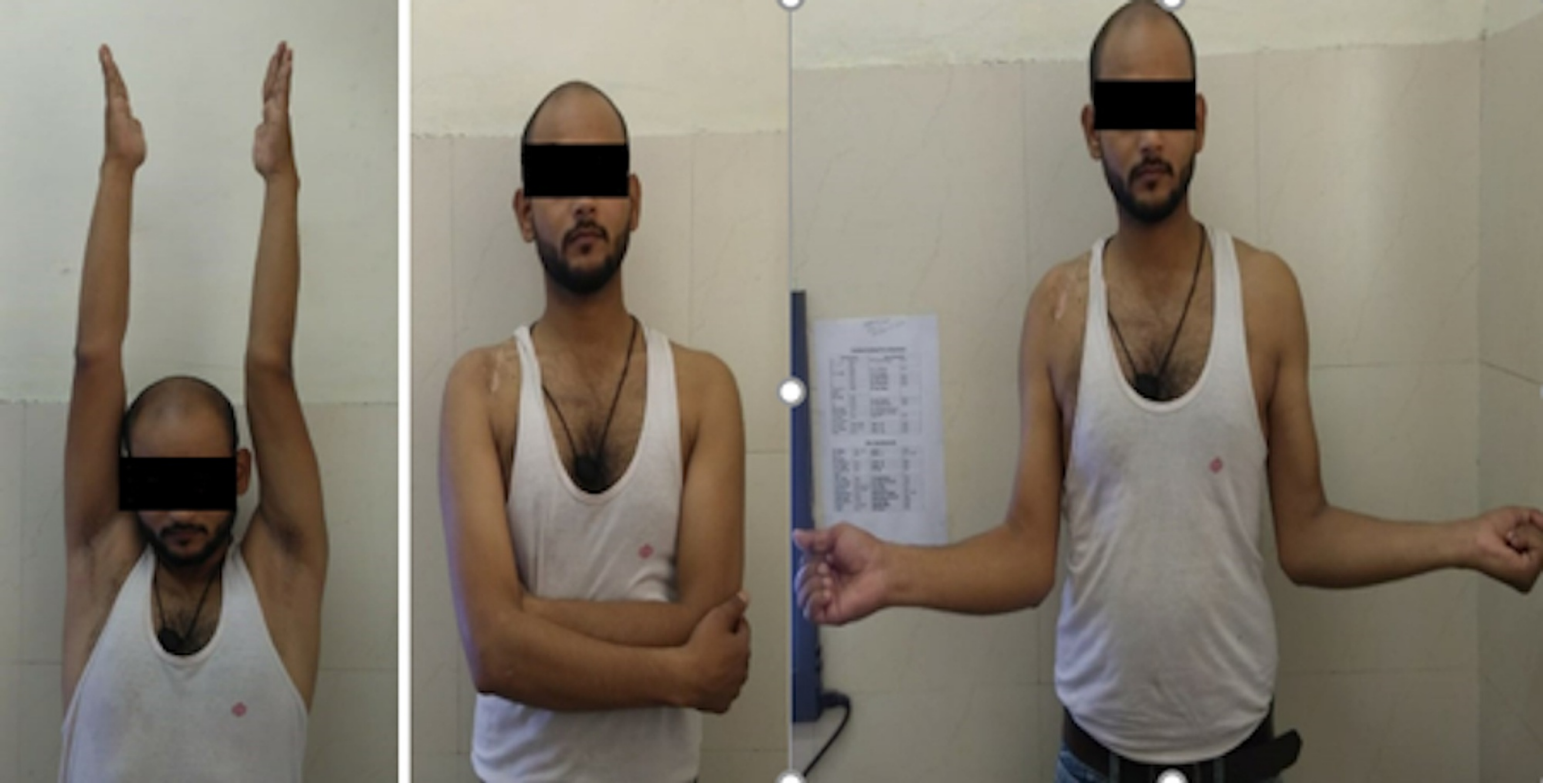
Range of motion of the shoulder at one-year follow-up.

The patients were followed at six weeks, three months, six months, one year, and thereafter every six months. At each visit, a thorough clinical assessment of each patient was done using the Constant Murley score and University of California Los Angeles (UCLA) score (Figure 3) [11,12]. 

**Figure 4:**
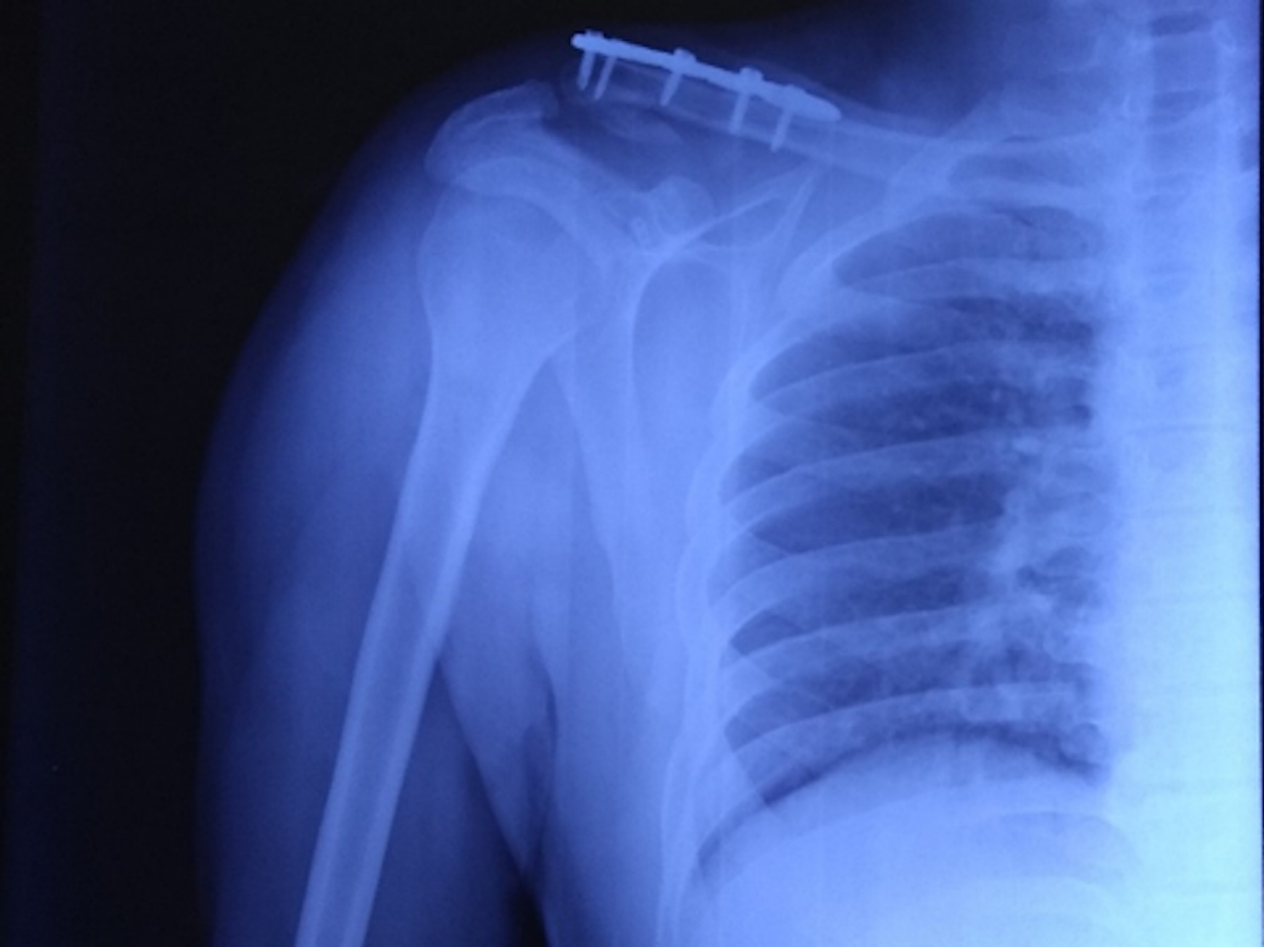
AP radiograph of the shoulder at one-year follow-up showing union of the lateral end of clavicle. AP: antero-posterior

Radiological assessment was also done at each visit with an anteroposterior radiograph with a 15-degree cephalic tilt (Zanca view) along with an axillary shoulder view to ascertaining the reduction of fracture fragments and position of the implant [13]. Contralateral shoulder radiographs were obtained in all cases for comparison. The coracoclavicular distance was measured on both normal and operated sides. Union at fracture site was confirmed radiologically by the continuity of cortex on three sides and medullary cavity reconstitution. Clinically, the absence of tenderness at the fracture site was considered as evidence of union (Figure 4).

All quantitative data were expressed as mean ± standard deviation. The clinical scores and radiological parameters were compared between the normal and operated shoulder at the latest follow-up. Statistical significance of differences in the mean values of continuous variables was determined using the Student t-test. Chi-square test was used for categorical variables. Fischer's exact test replaced the chi-square test when one of the cells in categorical values was zero. Statistical Package For The Social Sciences (SPSS) ver. 14.0 (SPSS Inc. Chicago, IL) was used for statistical analysis. A p-value less than 0.05 was considered to indicate statistical significance. All aspects of the statistical analysis were reviewed by a statistician.

## Results

Thirty-two consecutive patients with Neer type II fractures of the lateral end of clavicle were treated surgically using a pre-contoured locking distal clavicle plate along with coracoclavicular reconstruction with one endobutton and one No. 2 fiberwire. Out of these 32 patients, 22 were male and 10 were female. The average age of patients was 35.14±8.49 years. The mean age of male patients was 34.77±9.14 years while the mean age of female patients was 36±7.14 years. The dominant limb was involved in 26 patients (right 24, left 2) and the non-dominant limb was involved in six patients (right 2, left 4). The mean interval time between the injury and surgery was 5.26±2.18 days (range: three to nine days). The mean duration of the surgery was 56.23±7.24 minutes (range: 49-84 minutes) and the mean blood loss during the surgery was 158.26 ml (range: 90-200 ml). The mean time for the radiological union was 11±2.8 weeks (range: eight to 14 weeks). The mean follow-up period was 19±2.3 months (range: 13-35 months). The mean UCLA shoulder rating score, the mean Constant Murley score, and the mean coracoclavicular distance at different follow-up have been illustrated in Table 1. 

**Table 1:** Comparison of the serial functional and radiological outcome. UCLA: University of California Los Angeles

Parameters	Normal side	Injured side (3 months)	Injured side (6 months)	Injured side ( 1 year)	Injured side (latest follow-up)	P-value between injured side at latest follow-up and normal side
Mean UCLA shoulder rating score	34.14±1.76 (range 31-35)	28.94±1.68 (range 26-31)	30.44±2.26 (range 27-33)	32.18±1.82 (range 27-34)	33.54±2.01 (range29-35)	0.54
Mean Constant Murley score	96.16±2.01 (range 91-100)	79.91±3.55 (range 73-89)	86.88±3.12 (range 82-96)	92.23±2.98 (range 86-98)	94.84±3.02 (range 86-99)	0.58
Mean coracoclavicular distance (in mm)	10.24±0.76 (range 9.8-11.4)	10.38±0.84 (range 9.6-11.5)	10.44±0.98 (range 9.6-11.6)	10.58±0.76 (range 9.7-11.8)	10.62±0.88 (range 9.7-12.0)	0.28

**Figure 5:**
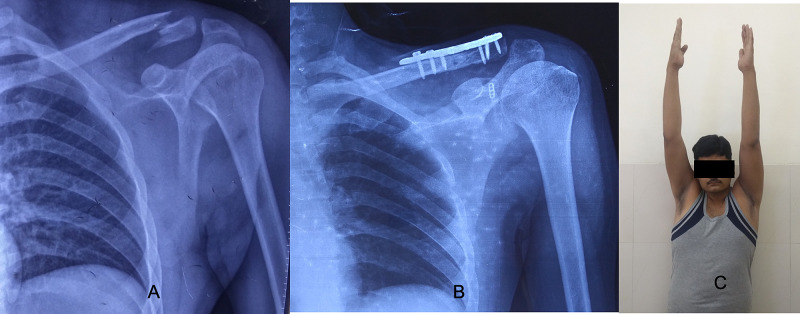
(A, B, C) Pre- and post-surgery radiograph and the clinical image showing the excellent result.

As per the Constant Murley scoring system, 29 (90.6%) cases had excellent results (Figure 5) and three (9.4%) had good results. None of the patients showed poor outcomes. All the 32 patients were able to return to their preinjury level of activity. The mean forward flexion at one year was 175.8 degrees (170-180), the mean abduction was 174.6 degrees (165-180), and the mean internal rotation was 84 degrees (80-90). The mean Constant Murley score and the UCLA score of the injured shoulder at the latest follow-up were comparable to the normal shoulder and not statistically significant with a p-value of 0.58 and 0.54, respectively. There was a continuous increase in the coracoclavicular distance at all follow-ups because of possible loosening of endobutton fixation with time, however, it was not significant when compared to the normal side at the latest follow-up.

There was no major complication in any of our cases. One of our patients developed a superficial infection which was managed with debridement and intravenous antibiotics. Bony union was achieved in all cases.

No patient complained of pain in their shoulder at one-year follow-up. None of our cases developed non-union, AC joint subluxation, plate pull out, or endobutton migration and required removal of the implant due to hardware prominence. Although one patient did complain of slight screw prominence, the discomfort was not to an extent that required implant removal. No patient showed signs of rotator cuff weakness, impingement, or glenohumeral instability. 

## Discussion

There are significant displacing forces at the fracture site in a Neer type II fracture of the lateral end of the clavicle which acts both in the horizontal and vertical direction. These forces include the weight of the arm, the scapular rotation, the pull of both pectoralis muscles, the latissimus dorsi, and the sternocleidomastoid muscles. Therefore, operative management has been the mainstay of treatment of these unstable lateral end clavicle fractures [3,14]. Conservative treatment of such fractures is generally associated with high rates of non-union [1,3,14]. A systematic review of lateral clavicle fractures published in 2010 revealed non-union rates as high as 33% in patients managed conservatively, while non-union rates were just 6% in those who had been managed operatively [15]. The use of a pre-contoured plate alone for fixation of these fractures only neutralizes the horizontal deforming forces, hence there are chances of plate pull out [16]. So, adding coracoclavicular reconstruction would neutralize the vertical deforming forces also, thereby minimizing the chances of plate pull out. Our current study demonstrates that excellent clinical outcomes can be achieved if Neer type II distal clavicle fractures are managed by pre-contoured distal clavicle plate along with coracoclavicular reconstruction using endobutton and No. 2 fiberwire since it neutralizes both horizontal and vertical deforming forces.

Brouwer et al. reported axial pull-out of locking screws from the lateral end clavicle fragment when non-augmented plates were used for fixation of these types of fractures [17]. When locking plates are used alone, there are chances of AC joint subluxation due to iatrogenic injury to the AC joint capsule [18]. In our study, there were no cases of AC joint subluxation and the mean coracoclavicular distance at the latest follow-up was comparable with the normal shoulder.

 Many techniques can be used for coracoclavicular reconstruction like coracoclavicular screws [2], endobutton fixation [6], or suture anchors [10]. They are either used alone or in combination with pre-contoured distal locking plates [5]. Harris et al. reported that if a bicortical purchase can be achieved, coracoclavicular screw fixation is the strongest reconstruction technique [19]. However, if only one cortex is engaged, the strength decreases by 50%. The placement of these screws is, however, technically more challenging and less reproducible [20]. The use of these screws can result in complications such as screw pull-out, breakage, screw head irritation, and infection. Robinson et al. used endobutton alone for the fixation of these fractures [6]. Out of 14 patients in their study, one case of fibrous union and one case of shoulder stiffness were reported. This can be attributed to the lack of stable fixation. In our study, all the cases achieved union and no patient developed shoulder stiffness. The possible reason may be that a locking plate provides stable and rigid fixation and when supported by coracoclavicular reconstruction, early ROM exercises can be started without the fear of implant pull out and thereby minimizing the chances of shoulder stiffness.

Friedman et al. in their study used only suture anchors for the fixation of unstable distal clavicle fracture and AC joint dislocation. However, they reported cases where there was suture anchor pull-out, and revision surgery was required [21]. In our study, there was not a single case of endobutton pullout or migration and none of the cases required revision surgery for non-union, implant failure, or hardware-related problems.

Morsy et al. compared osteosynthesis of unstable distal clavicular fractures with and without coracoclavicular ligament reconstruction [22]. In their study, fixation was done with T-shaped locking plates and coracoclavicular reconstruction was done with suture anchors. They reported better post-operative ROM in the group which had undergone coracoclavicular reconstruction with suture anchors. They attributed this to faster rehabilitation and earlier active exercise. In our study also, all the patients had excellent Constant score (mean 94.84).

Bhatia et al reported the clinico-radiological outcomes of acromioclavicular joint sparing and spanning implants in the surgical treatment of lateral clavicle fractures with coracoclavicular ligamentous disruption [23]. They used distal radius locking plates for fixation of fracture and endobutton device, suture anchors, or coracoid cerclage for coracoclavicular reconstruction. They concluded that clinical outcomes of both acromioclavicular joints sparing and spanning implants were favorable, however, a combination of the distal plate with coracoclavicular reconstruction resulted in a stable fixation and significantly lower re-operation rates. 

There are pieces of evidence in the literature regarding the excellent functional outcomes in a Neer type II distal clavicle fracture when the distal locking plate was combined with coracoclavicular reconstruction [22,23]. However, most of these studies have used suture anchors in combination with distal locking plates and have reported complications such as suture anchor pull-out which in some cases have required revision. Our study shows that the functional outcome of distal locking plate with coracoclavicular reconstruction using endobutton and fiberwire is comparable to suture anchors and complications are far less.

This study had few limitations. The sample size was small and a longer follow-up was required to assess for any articular changes. This was a single-center study and further multicentric studies with longer follow-up are required to assess the long-term functional outcome and reproducibility of this technique. 

## Conclusions

Neer type II fracture of the lateral end of the clavicle has both horizontal and vertical stress forces acting at the fracture site. The management requires neutralizing both these forces and allowing early mobilization of the shoulder.

The pre-contoured locking distal clavicle plate along with coracoclavicular reconstruction with endobutton and No. 2 fiberwire helps in counteracting both the stresses and provides excellent functional and radiological outcomes.
